# Estimating Implicit and Explicit Gender Bias Among Health Care Professionals and Surgeons

**DOI:** 10.1001/jamanetworkopen.2019.6545

**Published:** 2019-07-05

**Authors:** Arghavan Salles, Michael Awad, Laurel Goldin, Kelsey Krus, Jin Vivian Lee, Maria T. Schwabe, Calvin K. Lai

**Affiliations:** 1 Section of Minimally Invasive Surgery, Department of Surgery, Washington University in St Louis, St Louis, Missouri; 2 Medical student, School of Medicine, Washington University in St Louis, St Louis, Missouri; 3 Department of Psychological and Brain Sciences, Washington University in St Louis, St Louis, Missouri

## Abstract

**Importance:**

The Implicit Association Test (IAT) is a validated tool used to measure implicit biases, which are mental associations shaped by one’s environment that influence interactions with others. Direct evidence of implicit gender biases about women in medicine has yet not been reported, but existing evidence is suggestive of subtle or hidden biases that affect women in medicine.

**Objectives:**

To use data from IATs to assess (1) how health care professionals associate men and women with career and family and (2) how surgeons associate men and women with surgery and family medicine.

**Design, Setting, and Participants:**

This data review and cross-sectional study collected data from January 1, 2006, through December 31, 2017, from self-identified health care professionals taking the Gender-Career IAT hosted by Project Implicit to explore bias among self-identified health care professionals. A novel Gender-Specialty IAT was also tested at a national surgical meeting in October 2017. All health care professionals who completed the Gender-Career IAT were eligible for the first analysis. Surgeons of any age, gender, title, and country of origin at the meeting were eligible to participate in the second analysis. Data were analyzed from January 1, 2018, through March 31, 2019.

**Main Outcomes and Measures:**

Measure of implicit bias derived from reaction times on the IATs and a measure of explicit bias asked directly to participants.

**Results:**

Almost 1 million IAT records from Project Implicit were reviewed, and 131 surgeons (64.9% men; mean [SD] age, 42.3 [11.5] years) were recruited to complete the Gender-Specialty IAT. Healthcare professionals (n = 42 991; 82.0% women; mean [SD] age, 32.7 [11.8] years) held implicit (mean [SD] *D* score, 0.41 [0.36]; Cohen *d* = 1.14) and explicit (mean [SD], 1.43 [1.85]; Cohen *d* = 0.77) biases associating men with career and women with family. Similarly, surgeons implicitly (mean [SD] *D* score, 0.28 [0.37]; Cohen *d* = 0.76) and explicitly (men: mean [SD], 1.27 [0.39]; Cohen *d* = 0.93; women: mean [SD], 0.73 [0.35]; Cohen *d* = 0.53) associated men with surgery and women with family medicine. There was broad evidence of consensus across social groups in implicit and explicit biases with one exception. Women in healthcare (mean [SD], 1.43 [1.86]; Cohen *d* = 0.77) and surgery (mean [SD], 0.73 [0.35]; Cohen *d* = 0.53) were less likely than men to explicitly associate men with career (*B* coefficient, −0.10; 95% CI, −0.15 to −0.04; *P* < .001) and surgery (*B* coefficient, −0.67; 95% CI, −1.21 to −0.13; *P* = .001) and women with family and family medicine.

**Conclusions and Relevance:**

The main contribution of this work is an estimate of the extent of implicit gender bias within surgery. On both the Gender-Career IAT and the novel Gender-Specialty IAT, respondents had a tendency to associate men with career and surgery and women with family and family medicine. Awareness of the existence of implicit biases is an important first step toward minimizing their potential effect.

## Key Points

**Question:**

Do surgeons and health care professionals hold implicit or explicit biases regarding gender and career roles?

**Findings:**

A review of 42 991 Implicit Association Test records and a cross-sectional study of 131 surgeons provided evidence of implicit and explicit gender bias. Data suggest that health care professionals and surgeons hold implicit and explicit biases associating men with careers and surgery and women with family and family medicine.

**Meaning:**

This work contributes an estimate of the extent of implicit gender bias within medicine; awareness of bias, such as through an Implicit Association Test, is an important first step toward minimizing its potential effect.

## Teaser

This data review and cross-sectional study assesses how health care professionals associate men and women with career and family and how surgeons associate men and women with surgery and family medicine.

## Introduction

Enrollment of women in medical school has been nearly equivalent to that of men in the United States since 1999^1^ and has recently surpassed that of men for the first time.^2^ Despite this apparent equality, as of 2017 only 41% of all faculty and approximately 24% of full professors were women.^3^ These gaps are even larger when looking at department chairs: only 14% are women.^4^ Many factors likely contribute to women’s lack of equal representation in medical careers beyond medical school. Perhaps academic medical careers are less interesting or attractive to women than they are to men, or maybe pressures within medical training and academics favor men over women.

Implicit biases, or mental associations outside of conscious awareness or control that influence one’s interactions with others,^5^ may hinder the advancement of women in medicine. Sometimes, implicit biases lead people to act in ways that are not in line with their explicit beliefs or values.^6^ For example, one may explicitly believe that men and women are equally good at math. However, implicitly or unconsciously, one might be more likely to associate math with men than with women. These biases are shaped by the environment in which we live and are only weakly related to one’s conscious attitudes or beliefs. Importantly, implicit biases are associated with behaviors in socially sensitive contexts, such as interracial interactions.^7,8^

Direct evidence of implicit biases concerning women in medicine has not yet been reported, to our knowledge, but existing evidence is suggestive of subtle or hidden biases. For example, women physicians are often addressed as *Nurse* instead of *Doctor* or are introduced by their first name rather than their title.^9^ A study from 2016 showed that Medicare reimbursements to female physicians are lower than reimbursements to male physicians.^10^ When Silver et al^11^ tracked societal awards given out since 1945, they found that many societies had never given an award to a woman. Women are also less likely than men to be invited to give grand rounds, particularly as an outside speaker.^12^ One might argue that these discrepancies are due to women being less competent than men. However, these biases persist even in experiments in which candidates are matched on qualifications but differ in gender. For example, despite identical qualifications on a curriculum vitae, evaluators perceive male applicants to be more hirable and worthy of higher salaries than female applicants.^13^ Together, these data suggest that bias is an important factor that preempts women’s success in medicine.

The Implicit Association Test (IAT) was developed and validated to measure implicit biases^14^ and has demonstrated high internal consistency and robust evidence for predictive validity in numerous studies.^7,15,16^ To understand the degree of gender bias within the broad context of hospitals and health care systems, we examined the data of several thousand health care professionals who took the Gender-Career IAT from Project Implicit, the largest host of online IATs, with more than 26 million IATs started since 1998. Similar to how others have used IATs to assess health care professionals’ weight bias^17^ or associations of race with adherence,^18^ we developed a novel Gender-Specialty IAT to assess how surgeons associate men and women with surgery and family medicine. Surgery is of particular interest because of the known gender imbalance in the field, with only 25% of assistant professors being women.^19^ Previous data suggest that men and women in surgery perceive a gender ability stereotype to exist within this field.^20^ We chose family medicine as a comparison field because it may not be widely stereotyped as being masculine or feminine compared with other medical specialties. We hypothesized that men and women would be faster to associate men with surgery and women with family medicine than the reverse.

## Methods

We followed the Strengthening the Reporting of Observational Studies in Epidemiology (STROBE) reporting guideline for reporting cross-sectional studies. Use of the Gender-Career IAT data and recruitment for the Gender-Specialty IAT were approved by the institutional review board of Washington University in St Louis, St Louis, Missouri. Participants taking the Gender-Specialty IAT provided written informed consent.

In an IAT, people sort words that appear on the screen into categories as quickly as they can. Concepts that are closely associated should be easier to sort together quickly. For example, in the Gender-Career IAT, participants sort gender (*male* or *female*) and career (*career* or *family*). In 1 part of the Gender-Career IAT, participants sort words related to *male* or *career* to one side of the screen and words related to *female* or *family* to the opposite side. In the next part, they do the reverse: instead of *male/career* and *female/family* being sorted to the same side, *male/family* are sorted together, as are *female/career*. The test uses reaction times for these tasks as a measure of the strength of associations between concepts. Thus, if one is faster at pairing *male* with *career* and *female* with *family* than *male* with *family* and *female* with *career*, a stronger association for men with careers and women with families than the reverse is suggested.

### Gender-Career IAT

The Gender-Career IAT is hosted on the Project Implicit site and has been taken by 953 878 people during the past 12 years. From January 1, 2006, through December 31, 2017, 42 991 people who took the Gender-Career IAT self-identified as working in health care, and approximately one-fourth of these self-identified as diagnosing and treating professionals. The remaining categories of participants in health care are listed in Table 1. We downloaded the full data set, which is available from Project Implicit.^21^ In addition to the measure of implicit bias, the Gender-Career IAT included 2 questions assessing explicit bias: “How strongly do you associate career with males and females?” and “How strongly do you associate family with males and females?” Responses ranged from “strongly female” (1) to “strongly male” (7). As in previous IAT research, the measure of explicit bias was calculated as the difference between these 2 items, ranging from −6 (career is strongly female, whereas family is strongly male) to 6 (career is strongly male, whereas family is strongly female).^22^

**Table 1.** Descriptive Statistics for Health Care Professionals Taking the Gender-Career Implicit Association Test

Characteristic	Statistic (n = 42 991)^a^	Implicit Bias, Mean (SD), *D* Score^b^	Explicit Bias, Mean (SD)
Gender (n = 42 789)			
Male	7722 (18.0)	0.31 (0.39)	1.44 (1.79)
Female	35 067 (82.0)	0.44 (0.35)	1.43 (1.86)
Age, mean (SD), y (n = 32 834)	32.7 (11.8)	NA	NA
Race/ethnicity (n = 42 177)			
White	29 169 (69.2)	0.42 (0.36)	1.45 (1.75)
Black	4012 (9.5)	0.44 (0.37)	1.48 (2.33)
Asian	2895 (6.9)	0.36 (0.36)	1.49 (1.81)
Hispanic	3606 (8.5)	0.38 (0.37)	1.34 (1.98)
Other	2495 (5.9)	0.37 (0.37)	1.30 (1.87)
Educational attainment (n = 42 823)			
No high school degree	578 (1.3)	0.33 (0.38)	1.05 (2.07)
High school degree only or some college	11 563 (27.0)	0.42 (0.36)	1.40 (2.02)
Associate’s degree	6955 (16.2)	0.45 (0.35)	1.46 (2.00)
Bachelor’s degree	7649 (17.9)	0.41 (0.36)	1.43 (1.79)
At least some graduate school	16 078 (37.5)	0.40 (0.37)	1.45 (1.66)
Occupation (n = 42 991)			
Diagnosis and treatment of patients	10 718 (24.9)	0.37 (0.38)	1.50 (1.61)
Technologists and technicians	3556 (8.3)	0.41 (0.36)	1.46 (1.93)
Nursing and home health care assistants	14 404 (33.5)	0.44 (0.35)	1.41 (1.98)
Occupational and physical therapy assistants	1538 (3.6)	0.43 (0.36)	1.40 (1.80)
Other health care support	12 775 (29.7)	0.41 (0.36)	1.39 (1.87)
Country (n = 32 871)			
United States	29 803 (90.7)	0.43 (0.36)	1.58 (1.88)
Other	1850 (6.2)	0.38 (0.37)	1.04 (1.72)
Canada	615 (1.9)	0.40 (0.37)	1.62 (1.67)
United Kingdom	603 (1.8)	0.40 (0.38)	1.58 (1.56)

Abbreviation: NA, not applicable.

^a^ Unless otherwise indicated, data are expressed as number (percentage) of respondents. Percentages have been rounded and may not total 100.

^b^ Calculated by taking the difference in the mean reaction times for 2 sequences divided by the pooled SD.

### Gender-Specialty IAT Development

We developed an IAT with 2 categories (male and female) and 2 attributes (surgery and family medicine) based on the work of Greenwald and Banaji^5^ and the Gender-Career IAT available at Project Implicit.^23^ We replaced the terms for *career* and *family* with terms for *surgery* and *family medicine*. To ensure reliability of the IAT, stimuli must be accurate, clear, and similar across categories.^24^ Based on pilot data, we revised the terms for this study to make them even more evocative of surgery and family medicine. Initially chosen words, such as *scalpel* and *operating room,* could cause indecision for participants because they could be associated with ideas other than surgery. They also had no corresponding terms in family medicine. Professional organizations, on the other hand, are easy to recognize and could be matched to both specialties. Thus, we ultimately used logos from societies such as the American Board of Surgery and the American College of Surgeons. eTable 1 in the Supplement shows the terms and images used for surgery and family medicine as well as the test blocks. The names of men and women we used were the ones used in the Gender-Career IAT (Ben, John, Daniel, Paul, Jeffrey, Julia, Michelle, Anna, Emily, and Rebecca). The order of the blocks was randomly assigned so that some participants were first asked to associate *male* with *surgery* and *female* with *family medicine,* whereas others were first asked to associate *male* with *family medicine* and *female* with *surgery*. The IAT was run from the Project Implicit website^21^ with support from Project Implicit.

At the completion of the IAT, participants were asked questions similar to those on the Gender-Career IAT to assess their explicit bias about gender. One read as follows: “How strongly do you associate surgery with males and females?**”** with responses ranging from “strongly female” (1) to “strongly male” (7). Similar to the Gender-Career IAT, a parallel question was asked about family medicine. Explicit bias was calculated as the difference between these 2 items, ranging from −6 (surgery is strongly female, whereas family medicine is strongly male) to 6 (surgery is strongly male, whereas family medicine is strongly female). Participants were also asked demographic questions, including gender, race, title, country, and region. For ease of data collection, data were collected using tablet devices.

### Participants

We collected data from the Gender-Career IAT on Project Implicit and focused most analyses on participants who work in health care fields. For the novel Gender-Specialty IAT, we recruited surgeons (in practice and in training) in attendance at the American College of Surgeons meeting in October 2017 in San Diego, California. They were recruited by volunteers throughout meeting hotels and the convention center. Participants received a $10 Amazon gift card in exchange for their participation.

### Statistical Analysis

Data were analyzed from January 1, 2018, through March 31, 2019. The IAT is scored using the *D* score, a measure of bias based on the reaction times in the experimental blocks of the test (sequences 3 and 5 in eTable 1 in the Supplement).^15^ The *D* score is a variation on the Cohen *d* and is calculated by taking the difference in the mean reaction times for those 2 sequences divided by the pooled SD. The *D* scores range from −2 to 2, with positive *D* scores indicating a stronger association of men with career (or surgery) and women with family (or family medicine) and negative scores indicating the reverse. *D* scores are roughly equivalent in interpretation to the Cohen *d*, with a *D* score of 0.50 meaning that a participant was 0.5-SD faster in responding to men and career (or surgery) and to women and family (or family medicine) than the reverse. The means reported for the implicit IAT measure as well as those used in regression analyses for that measure are the means of the *D* scores.

The *D* score is a within-participants effect size comparing differences between one’s reaction times in 2 IAT blocks. The Cohen *d*, by contrast, is an effect size comparing the within-participant effect size with an external standard (eg, the point of no preference or a group mean). Thus, although the *D* score is an estimate of the difference in response times between blocks on the IAT, the Cohen *d* is an estimate of how different that score is from the point of no preference (in a single-sample test) or how different the scores of 2 different groups are from each other (when comparing means of 2 groups). As is common, we interpret effect sizes of approximately 0.2 to be small, approximately 0.5 to be medium, and approximately 0.8 or greater to be large.^25^

For the Gender-Career IAT, we examined the overall *D* scores for health care professionals as well as differences in the *D* score and the measure of explicit bias by type of worker. We also analyzed differences in implicit and explicit bias by gender, age, and region.

We performed similar analyses for our novel Gender-Specialty IAT. Participants received feedback on their performance at the end of the IAT. We analyzed the *D* scores to assess the overall mean as well as any differences by gender, title, or region.

For both IATs, we used 2-tailed *t* tests for comparisons between 2 groups and analysis of variance for comparisons among multiple groups. We used linear regression analyses while controlling for demographic variables to examine associations between those variables and implicit and explicit bias. The threshold for statistical significance was set a priori at 2-sided α = .05 for all statistical analyses. All analyses were performed in SAS, version 9.4 (SAS Institute Inc). Only complete responses were included in analyses.

## Results

### Implicit Gender-Career Bias

A total of 42 991 health care professionals completed the Gender-Career IAT (Table 1). Consistent with the health care workforce, 82.0% of respondents were women, and 18.0% were men. Mean (SD) age was 32.7 (11.8) years. Most participants (69.2%) were white. A little more than one-third (33.5%) were nursing and home health care assistants, and 24.9% were diagnosing and treating professionals. Data were also available from 910 887 participants who were not health care professionals (67.5% female and 68.3% white).

The IAT scores linking men with career and women with family were significantly different from zero among health care professionals (mean [SD] *D* score, 0.41 [0.36]; Cohen *d* = 1.14) and non–health care professionals (mean [SD] *D* score, 0.37 [0.38]; Cohen *d* = 0.97). Health care professionals exhibited slightly stronger implicit associations for men with career and women with family than non–health care professionals (*t*_46,921_ = −23.65; *P* < .001; Cohen *d* = 0.11). Interestingly, female (mean [SD] *D* score, 0.44 [0.35]; Cohen *d = *1.23) and male (mean [SD] *D* score, 0.31 [0.39]; Cohen *d* = 0.79) health care professionals exhibited implicit associations of men with career and women with family that were significantly different from zero. These associations were stronger among female health care professionals than among male health care professionals (*t*_10,621_ = 26.89; *P* < .001; Cohen *d* = 0.35). A significant difference was evident among the categories of health care professionals such that diagnosing and treating professionals whose scores were significantly different from zero (mean [SD] *D* score, 0.37 [0.38]; Cohen *d* = 0.97) showed significantly lower scores than each of the other categories (*t* ≤ −4.76; *P* < .001 for all pairwise comparisons).

In regression analyses of implicit bias from gender, age, ethnicity, and country, we found that women were slightly more likely than men to associate men with career and women with family (*B* coefficient, 0.13; 95% CI, 0.12-0.14; *P* < .001). Other statistically significant findings are given in Table 2, such as the findings related to age, race, and country of residence. However, the regression coefficients are so small that these findings are not practically significant.

**Table 2.** Regression Analysis of Implicit and Explicit Bias From the Gender-Career Implicit Association Test

Independent Variable	Implicit Bias			Explicit Bias		
	*B* Coefficient (95% CI)	*t* Statistic^a^	*P* Value	*B* Coefficient (95% CI)	*t* Statistic^a^	*P* Value
Gender						
Male	1 [Reference]	NA	NA	1 [Reference]	NA	NA
Female	0.13 (0.12 to 0.14)	24.90	<.001	−0.10 (−0.15 to −0.04)	−3.52	<.001
Age	0.002 (0.00 to 0.00)	9.35	<.001	−0.002 (−0.00 to −0.00)	−2.24	.03
Race/ethnicity						
White	1 [Reference]	NA	NA	1 [Reference]	NA	NA
Black	0.01 (−0.00 to 0.03)	1.82	.07	−0.04 (−0.11 to 0.03)	−1.05	.29
Asian	−0.03 (−0.05 to −0.02)	−3.89	<.001	0.03 (−0.05 to 0.12)	0.72	.47
Hispanic	−0.03 (−0.05 to −0.02)	−4.25	<.001	−0.11 (−0.18 to −0.03)	−2.72	.007
Other	−0.04 (−0.06 to −0.03)	−5.25	<.001	−0.18 (−0.26 to −0.09)	−3.96	<.001
Residence						
United States	1 [Reference]	NA	NA	1 [Reference]	NA	NA
Canada	−0.03 (−0.05 to 0.00)	−1.70	.09	0.02 (−0.13 to 0.17)	0.30	.77
Other	−0.01 (−0.03 to 0.00)	−1.36	.17	0.10 (0.02 to 0.17)	2.37	.02

Abbreviation: NA, not applicable.

^a^ Indicates 2-tailed *t* test.

### Explicit Gender-Career Bias

Explicit bias responses associating men with career and women with family were significantly different from zero for both women (mean [SD], 1.43 [1.86]; Cohen *d* = 0.77) and men (mean [SD], 1.44 [1.79]; Cohen *d* = 0.80) in health care (*t*_11,585_ = −0.64; *P* = .52, Cohen *d* = −0.01 for the comparison by gender). Explicit bias was significantly different from zero among health care professionals (mean [SD], 1.43 [1.86]; Cohen *d* = 0.77) and non–health care professionals (mean [SD], 1.36 [1.73]; Cohen *d* = 0.79). Health care professionals exhibited more explicit bias than non–health care professionals (*t*_46,554_ = −7.23; *P* < .001; Cohen *d* = 0.04). All categories of health care professionals expressed explicit bias linking men with career and women with family, including diagnosing and treating professionals (mean [SD], 1.50 [1.61]; Cohen *d = *0.93), nursing and home health care assistants (mean [SD], 1.41 [1.98]; Cohen *d* = 0.71), and other health care support (mean [SD], 1.39 [1.87]; Cohen *d* = 0.74). When we compared categories of health care professionals, those professionals who were diagnosing and treating patients were more likely to explicitly associate men with career and women with family than were nursing and home health care assistants (*t*_24,717_ = 4.06; *P* < .001; Cohen *d* = 0.05) and other health care support (*t*_23,298_ = 5.07; *P* < .001; Cohen *d* = 0.06).

In contrast with the regression analysis of implicit bias, Table 2 demonstrates that women were less likely than men to express an explicit association of men with career and women with family (*B* coefficient, −0.10; 95% CI, −0.15 to −0.04; *P* < .001). Hispanic participants and participants of other races/ethnicities were less likely than white participants to explicitly associate men with career and women with family (Hispanic participants: *B* coefficient, −0.11 [95% CI, −0.18 to −0.03]; *t*_32,009_ = −2.72; *P* = .007; participants of other races/ethnicities: *B* coefficient, −0.18 [95% CI, −0.26 to −0.09]; *t*_32,009_ = −3.96; *P* < .001).

### Implicit Gender-Specialty Bias

We collected complete data on the Gender-Specialty IAT from 131 participants. Table 3 provides the demographic characteristics of the participants in the study. Eighty-five participants (64.9%) were men and 45 (34.4%) were women. The mean (SD) age of these participants was 42.3 (11.5) years, and 77 (58.8%) were white. Participants were distributed across all titles (assistant professor, associate professor, and full professor).

**Table 3.** Descriptive Statistics for the Gender-Specialty Implicit Association Test

Characteristic	Statistic (n = 131)^a^	Implicit Bias, Mean (SD), *D* Score^b^	Explicit Bias, Mean (SD)
Gender			
Male	85 (64.9)	0.28 (0.39)	1.27 (0.39)
Female	45 (34.4)	0.27 (0.35)	0.73 (0.35)
Other	1 (0.8)	0.05 (NA)	0.00 (NA)
Age, mean (SD), y	42.3 (11.5)	NA	NA
Race/ethnicity			
White	77 (58.8)	0.31 (0.40)	0.94 (1.30)
Black	15 (11.5)	0.30 (0.42)	1.20 (1.37)
Asian	24 (18.3)	0.24 (0.33)	1.42 (1.64)
Hispanic	10 (7.6)	0.12 (0.32)	1.10 (1.45)
Other	5 (3.8)	0.21 (0.13)	1.20 (1.64)
Title			
Assistant professor	25 (19.1)	0.33 (0.37)	1.24 (1.23)
Associate professor	18 (13.7)	0.38 (0.41)	1.00 (1.08)
Professor	17 (13.0)	0.30 (0.37)	1.29 (1.53)
Private practice	21 (16.0)	0.18 (0.36)	0.62 (1.56)
Other	50 (38.2)	0.28 (0.38)	1.14 (1.44)
Country			
United States	98 (74.8)	0.29 (0.40)	0.84 (1.27)
Canada	11 (8.4)	0.33 (0.39)	1.45 (1.21)
Other	22 (16.8)	0.19 (0.21)	1.95 (1.62)
Region^c^			
Northeast	20 (23.8)	0.18 (0.40)	1.10 (1.37)
Midwest	17 (20.2)	0.31 (0.34)	1.24 (1.25)
South	28 (33.3)	0.33 (0.41)	0.79 (1.17)
West	19 (22.6)	0.38 (0.42)	0.26 (0.56)

Abbreviation: NA, not applicable.

^a^ Unless otherwise indicated, data are expressed as number (percentage) of respondents. Percentages have been rounded and may not total 100.

^b^ Calculated by taking the difference in the mean reaction times for 2 sequences divided by the pooled SD.

^c^ Includes 84 respondents within the United States.

The mean IAT score indicated a significant association linking men with surgery and women with family medicine (mean [SD] *D* score, 0.28 [0.37]; Cohen *d* = 0.76). No difference in IAT scores was found between male and female participants (*t*_99.04_ = −0.11; *P* = .91; Cohen *d* = −0.03). When we restricted data to those living in the United States, no significant difference in gender bias was found by region (*F*_3,80_ = 0.89; *P* = .45).

None of the demographic variables we collected correlated with implicit bias. As shown in eTable 2 in the Supplement, no demographic variables were statistically significant in a regression analysis of implicit bias from gender, age, race, and title.

### Explicit Gender-Specialty Bias

Explicit bias responses associating men with surgery and women with family medicine were significantly different from zero for men (mean [SD], 1.27 [0.39]; Cohen *d* = 0.93) and women (mean [SD], 0.73 [0.35]; Cohen *d* = 0.53). Men expressed more explicit bias than did women (*t*_88.50_ = −2.11; *P* = .04; Cohen *d* = 0.39).

As shown in eTable 2 in the Supplement, regression analysis of the explicit bias measure from gender, age, race, and title found that women were less likely than men to associate men with surgery and women with family medicine (*B* coefficient, −0.67; 95% CI, −1.21 to −0.13; *P* = .001). Those in private practice also were less likely to associate men with surgery and women with family medicine than those who had listed their title as “other” (*B* coefficient, −1.13; 95% CI, −1.96 to −0.29; *P* = .009). Those who identified as Asian were more likely than white participants to associate men with surgery and women with family medicine (*B* coefficient, 0.81; 95% CI, 0.13-1.48; *P* = .02). There was no difference in explicit bias in surgery by age (*B* coefficient, 0.02; 95% CI, −0.01 to 0.05; *P* = .26).

**Figure 1.**
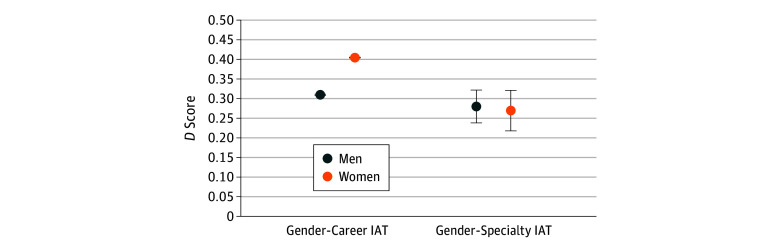
Implicit Association Test (IAT) Standardized *D* Scores by Participant Gender For the Gender-Career IAT, implicit measures include 34 662 women and 7624 men; explicit measures, 34 835 women and 7675 men. For the Gender-Specialty IAT, implicit and explicit measures included 45 women and 85 men. Error bars represent SE. Standard errors for the Gender-Career IAT data are so small that they are not visible on the graph.

**Figure 2.**
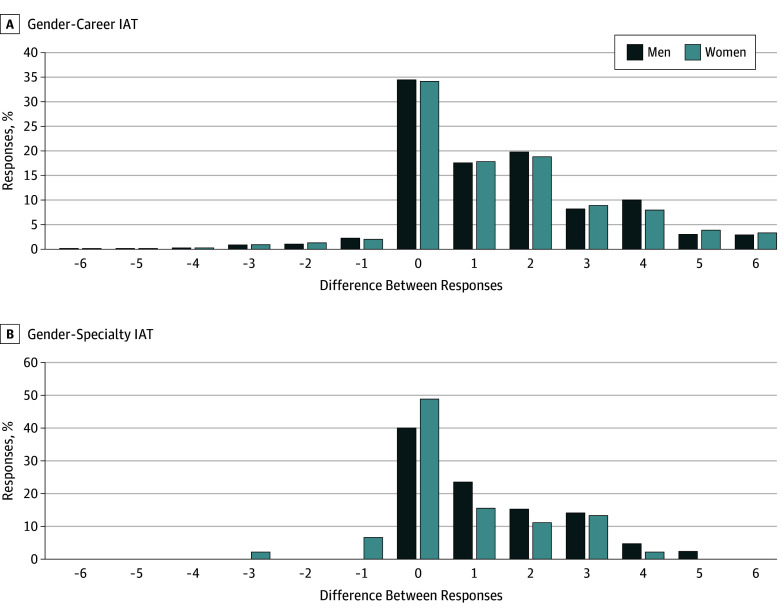
Explicit Bias Scores by Participant Gender Explicit bias scores are calculated as the difference between the responses to 2 self-reported items about participants’ associations of gender with career and family (Gender-Career Implicit Association Test [IAT]) or with surgery and family medicine (Gender-Specialty IAT).

Figure 1 and Figure 2 show differences between men and women on levels of implicit and explicit bias. These figures illustrate the finding that women expressed lower levels of explicit gender bias than did men. Data for the implicit measures were mixed, with women expressing slightly higher levels of implicit bias than men on the Gender-Career IAT, whereas no difference by gender was noted on the Gender-Specialty IAT.

## Discussion

The data from Project Implicit’s Gender-Career IAT suggest that men and women in health care strongly implicitly associate men with career and women with family. With regard to explicit bias, however, men in health care were more likely than women to associate men with career and women with family. These findings are similar to what we found with the Gender-Specialty IAT assessing bias among surgeons. Surgeons tended to associate men with surgery and women with family medicine. Thus, from both data sets we found that, although men and women associated men with career and surgery (and women with family and family medicine), men were more likely than women to consciously express a bias linking men with career or surgery and women with family or family medicine. Future research should replicate these findings and assess whether these biases are linked to existing gender disparities. For example, previous studies^26,27,28^ suggest that women may be more likely than men to leave surgical residency, and implicit gender biases could play a role. Girod et al^29^ have also suggested that implicit bias among senior faculty may contribute to the gender disparity in leadership roles in academic medicine.

On the novel implicit measure of gender bias about surgery and family medicine, we found evidence of consensus. Across all social categories assessed (gender, race, title, region of the United States, and country of origin), participants taking our novel Gender-Specialty IAT expressed implicit and explicit bias about men and women in surgery. We found that male and female surgeons’ implicit gender-specialty biases were large and similar in magnitude to male and female health care workers’ implicit gender-career biases. With explicit biases, we found evidence of a difference between genders. Explicit gender-specialty biases for male surgeons were large and similar in magnitude to explicit gender-career biases for male health care workers. However, explicit gender-specialty biases for female surgeons were smaller than explicit gender-career biases for female health care workers. This difference could be due to variation in sample populations or topics assessed. These data, although not definitive, suggest that biases linking surgery with men and family medicine with women may be widespread across the United States among surgeons. Unlike the Gender-Career IAT, we did not identify a difference between the genders in implicit bias on the Gender-Specialty IAT. However, this finding may be due to the smaller sample size in the Gender-Specialty IAT.

Diversity is important to the success of organizations.^30,31^ Specifically, organizations with more diverse leadership are more productive and profitable. Patients, who come from many different backgrounds, are more satisfied with their care when it is provided by someone who looks like them.^6,32^ Given that women are approximately 50% of the population and that an increasing percentage in the United States is of minority race or ethnicity, we must ensure that we foster physicians of all gender and racial groups. Having diverse people in leadership positions ensures that role models and potential mentors are available for all applicants.^33^ Role models and mentors, in turn, are important for recruiting trainees who are members of underrepresented groups.^34^ To improve recruitment and retention of diverse trainees, we need to better understand the factors that contribute to underrepresentation of women.

For many, awareness of bias is an important first step toward minimizing its effects.^35^ The data presented herein help to raise awareness of gender bias within medicine. In addition, these data allow trainees to understand the context in which they will practice, thus better preparing them for their future work environment. Finally, this study adds to the existing evidence that organizations can use to make the case for prioritizing diversity and possibly implicit bias training.

### Limitations

This study lacks granularity about health care fields from the Gender-Career IAT data. Thus, we are not able to isolate, for example, physicians exclusively. The category of diagnosing and treating professionals may include dentists, nurse practitioners, and physician assistants, for example. In addition, selection bias for the Gender-Career IAT may lead to a lower estimate of the degree of bias present generally. As many as 0.43% of respondents may have been repeated sessions. Data from these IATs do not allow us to assess the effect of intersectionality or other genders, since both IATs focused on male/female gender alone. We cannot determine whether those sessions were different individuals using the same computer or the same individual.

A limitation of the novel Gender-Specialty IAT is that we recruited participants attending a surgical meeting. We appear to have undersampled older surgeons. This limitation is unlikely to affect the results dramatically because results on the Gender-Career IAT and other similar IATs found only small correlations between age and implicit biases.^22^ If anything, having fewer older surgeons may underestimate the degree of gender bias in this context. Our sample size for the novel Gender-Specialty IAT is modest.

## Conclusions

The main contribution of this work is an initial estimate of the extent of implicit gender bias within health care. Future research could examine implications of implicit gender biases on gender inequality and discrimination. Other research already provides some interventions for addressing gender bias regardless of whether it comes from implicit bias or other sources. For example, increasing transparency of hiring and promotion policies, considering diversity as a performance metric for organizations, and promoting flexible leave all serve to increase the success of female physicians and trainees.^36,37,38^ Further documentation of implicit associations and other potential psychological obstacles to women’s success will be important for determining the most effective interventions to reduce gender inequality. It is important to also intentionally study the effects of bias on individuals who hold more than one minority identity, such as black or Hispanic women. Such research will benefit current medical students who will become our physicians tomorrow.

## Notes

Supplement. **eTable 1.** IAT Design for Gender and Surgery vs Family Medicine **eTable 2.** Regression Analysis Predicting Implicit and Explicit Bias From the Gender-Specialty IAT
