# Supplementary material for: Estimating Implicit and Explicit Gender Bias Among Health Care Professionals and Surgeons
Source: JAMA Netw Open. 2019 Jul 5;2(7):e196545. doi: 10.1001/jamanetworkopen.2019.6545 (PMC12064094; doi:10.1001/jamanetworkopen.2019.6545)
Supplement: Supplement. — eTable 1. IAT Design for Gender and Surgery vs Family Medicine eTable 2. Regression Analysis Predicting Implicit and Explicit Bias From the Gender-Specialty IAT [file jamanetwopen-e196545-s001.pdf]

Supplementary Online Content

Salles A, Awad M, Goldin L, et al. Estimating implicit and explicit gender bias among health care professionals and surgeons. *JAMA Netw Open.* 2019;2(7):e196545. doi:10.1001/jamanetworkopen.2019.6545

eTable 1. IAT Design for Gender and Surgery vs Family Medicine

eTable 2. Regression Analysis Predicting Implicit and Explicit Bias From the Gender-Specialty IAT

This supplementary material has been provided by the authors to give readers additional information about their work.

© 2019 Salles A et al. *JAMA Network Open*

eTable 1. IAT Design for Gender and Surgery vs Family Medicine

Sequence	1	2	3*	4	5*
Task Description	Initial target-concept discrimination	Associated attribute discrimination	Initial combined task	Reversed target-concept discrimination	Reversed Combined Task
Task Instructions	Male<br>Female	Surgery<br>Family Medicine	Male/Surgery<br>Female/Family Medicine	Male<br>Female	Female/Surgery<br>Male/Family Medicine
Sample Stimuli	Julia<br>Michelle<br>Anna<br>Emily<br>Rebecca<br>Ben<br>John<br>Daniel<br>Paul<br>Jeffrey	Surgery<br>Surgeon<br>Family Medicine<br>Family Doctor	• Male names with surgeon targets<br>• Female names with family medicine targets	Julia<br>Michelle<br>Anna<br>Emily<br>Rebecca<br>Ben<br>John<br>Daniel<br>Paul<br>Jeffrey	• Female names with surgeon targets<br>• Male names with family medicine targets

*Blocks 3 and 5 are repeated.

Note. There are 20 trials in Blocks 1, 2, and the first iteration of Blocks 3 and 5, 28 trials in Block 4, and 40 trials in the second iteration of Blocks 3 and 5.

© 2019 Salles A et al. *JAMA Network Open*

eTable 2. Regression Analysis Predicting Implicit and Explicit Bias From the Gender-Specialty IAT*

		Implicit bias					Explicit bias		
**Independent variable**	***B***	***95% CI***	***t***	***p***		***B***	***95%CI***	***t***	***p***
Gender (ref = male)									
Female	-0.04	(-0.19, 0.12)	-0.47	0.64		-0.67	(-1.21, -0.13)	-2.45	0.02
Age	-0.002	(-0.01, 0.01)	-0.47	0.64		0.02	(-0.01, 0.05)	1.14	0.26
Race/ethnicity (ref = White)									
Black	0.01	(-0.21, 0.22)	0.06	0.95		0.37	(-0.40, 1.14)	0.96	0.34
Asian	-0.06	(-0.25, 0.13)	-0.59	0.55		0.81	(0.13, 1.48)	2.36	0.02
Hispanic	-0.18	(-0.44, 0.08)	-1.40	0.16		0.22	(-0.70, 1.13)	0.47	0.64
Other	-0.11	(-0.46, 0.25)	-0.59	0.56		0.52	(-0.75, 1.79)	0.81	0.42
Title (ref = other)									
Assistant professor	0.06	(-0.13, 0.26)	0.64	0.52		-0.04	(-0.75, 0.66)	-0.13	0.90
Associate professor	0.01	(-0.23, 0.25)	0.10	0.92		-0.60	(-1.45, 0.26)	-1.39	0.17
Professor	0.04	(-0.25, 0.33)	0.28	0.78		-0.53	(-1.56, 0.51)	-1.00	0.32
Private practice	-0.08	(-0.31, 0.16)	-0.64	0.52		-1.13	(-1.96, -0.29)	-2.68	0.009

**B*=regression coefficient; CI=confidence interval; *t*=*t*-statistic; *p*-value

© 2019 Salles A et al. *JAMA Network Open*
